# The Week's Work

**Published:** 1910-05-28

**Authors:** 


### The Week's Work.

### A NEW HOSPITAL AND STATE INSURANCE.

Dr. De Voogt, for many years chief of the medical staff at the Gouda Hospital, contributes an article to *Het Ziekenhuis* on the new Van Iterson Hospital at Gouda. This institution was founded by the Mesdames van Iterson in memory of their father, and is a model hospital of 76 beds, built on such a plan that it can later on be added to and extensively improved if necessary. For instance, at present arrangements have been made for lighting the wards by gaslight, but an electric installation can soon be added when the institution possesses, as it hopes to possess very shortly, its own electric plant. So, too, the lifts are at present hand lifts, but they can easily be changed to automatic electric lifts of the usual model found in modern hospitals. The institution, which is under municipal control, deals with three classes of patients, the first class paying 4 florins (12 florins equals £1), the second 2 florins, and the third 1 florin. The third class is admitted free of charge on producing a certificate from the Burgerlyk Armbestuur (Civil Poor Authority) that the patient is too poor to pay the florin. Patients belonging to the Government Insurance Bank pay a florin a day nursing fee. All paying patients have the right of calling in their own physicians or surgeons, but in that case they must pay the cost of medical treatment. They may make use of the medical staff of the institution without additional charge, the members of the staff being paid. All the nurses are properly trained and certificated, and the management of the hospital is in every way up to date. The article is illustrated with blocks showing the ground plan and perspective view of the hospital. In the same number Dr. H. P. Bosscha continues his interesting series of articles on "The Hospital and Society," dealing in this one with the influence of the social insurance laws. Briefly, Dr. Bosscha shows that the system of insurance against sickness has had, both in Germany and in Holland, marked influence upon the hospitals. He deals with the dangers to the voluntary hospital system which such insurance scheme may produce, and how they may be averted. One of the most serious matters in connection with private insurance societies, so far as hospitals are concerned, is the tendency which these large associations show, as soon as their membership increases as soon as their sick fund grows, to start their own hospitals. In England this has already been done in the case of sanatoria, and on the Continent the tendency has been towards creating special institutions under the undivided control of such associations. Dr. Bosscha, however, thinks that this danger is dying out, and that in future such associations will co-operate loyally with existing hospitals so long as they are allowed a voice in the management of the institutions to which they contribute. In the case where there is State insurance the relations between the hospitals and the contributing body become more complex. The article is exceedingly ably written, and full statistics are given. Yet a third interesting contribution which appears in this number is that of Dr. Wortman on "Cottage Hospitals, their Construction and Equipment." This series deals exhaustively with the cottage model, and is specially full on the matter of apparatus, although, as is only fair, preference is given to types manufactured by Dutch and German firms, whose machinery is but little known in this country.

### A HISTORY OF ST. GEORGE'S HOSPITAL.

An extremely ambitious and comprehensive history of St. George's Hospital from its foundation in 1733 down to the close of the nineteenth century has long been in course of preparation by Mr. G. C. Peachey, M.R.C.S., an old *alumnus* of that school. From the announcement of the scheme which has just been published it is evident that, when complete, this history will eclipse most other records of the same nature, and will mark an important advance in hospital historical literature. The work is projected in twelve sections at a cost of half-a-crown each, and it is to deal with many topics which are of general interest to the hospital world as well as those more especially concerned with the institution at Hyde Park Corner; the first part will be ready for publication about the end of May. Of the four main sections into which the entire work is to be divided, one deals with the foundation of the hospital, and contains a long and important critical *résumé* of the origin and evolution of hospitals and of medical schools in England. The first part of this first section will contain an epitome of the provision made for the sick poor in England from early times down to the eighteenth century. The second part is to deal with Lanesborough House and its surroundings at the date of the transformation into St. George's Hospital, with illustrations from contemporary prints and pictures. Succeeding parts will take up in detail the history, management, staff, and other aspects of the hospital and its history; these will necessarily be less interesting to the general reader than to old St. George's men and nurses. But it is evident that the author has accumulated materials which form a valuable contribution to the history of the voluntary hospital movement in England, and that his many years of research have yielded him what promises to be a very notable addition to its literature. The work is issued with the approval of a sub-committee appointed to further the project of a history of the hospital, and the names of such well-known St. George's men as Dr. G. F. Blandford, Mr. C. T. Dent, and Mr. Warrington Howard are sufficient guarantee that the result will be worthy of the subject. It is to be hoped that some similar enterprises may be set on foot by patriotic sons of other ancient and notable hospitals in Great Britain. Such a work, to be of permanent value, demands, of course, much time and trouble, more, perhaps, than most men in active practice can give; but there must be many who have retired from the grind of daily medical work to well-earned leisure, yet have the experience and the mental and bodily vigour required for the undertaking of such tasks. If Mr. Peachey's labours spur on others to bring honour in a similar way upon their old hospitals he will be doubly rewarded. The success of his experiment is, we should imagine, almost assured already, and we trust in due time to be able to congratulate him on a full subscription list as well as the less substantial but even more gratifying encomiums which he deserves.

### A FRENCH HOSPITAL SCANDAL.

Considerable discussion has been aroused in the French Press by the revelations in the Candide case. It appears that Sister Candide, an ex-nun, who has been singularly active in connection with gathering subscriptions for many local charities, notably those catering for tuberculous patients, organised a series of lotteries in aid of the institutions in which she was interested. Of all the methods available for obtaining money for hospital purposes the lottery method is perhaps the least satisfactory that can be devised, and Sister Candide appears to have found it so, for her ventures proved failures and left her saddled with debt. The immediate cause of the revelations concerning the management of the charities was the action brought against the ex-nun by a firm of jewellers who alleged that she had pawned certain goods supplied to her on approval, and the public interest in the case has been hugely whetted by the statement that M. Emile Loubet has been connected with the affair. M. Loubet's sole share in the scandal seems to be the fact that he is honorary patron of one of the institutions taken over by Sister Candide, the sanatorium for children at San Salvador. Years ago he visited this institution and was so much impressed by its usefulness that he has since supported it, although he was unaware that shortly after his visit the institution passed into other hands and became the sole property of the ex-nun. He merely knew of her as a profoundly charitable person whom he had been asked to decorate as a slight mark of recognition for her meritorious services on behalf of charity in France. That these services have been meritorious there can be no question. It is probable that the sister's present embarrassment is due to her ignorance of business methods and to the fact that she has been systematically imposed upon by people who have taken advantage of her generosity and business incapacity. The public discussion which the affair has aroused is likely to prove of real service to French hospitals which exist upon the voluntary system—and these are mainly homes and sanatoria —in so far that it will force them to give an account of their expenditure and income on lines similar to that which every reputable charity adopts in this country. A great many small institutions on the Continent manage to exist, and even to flourish, without issuing balance sheets or holding annual meetings.

### CONSUMPTIVE PATIENTS IN GENERAL WARDS.

In how far is the retention of a patient obviously suffering from advanced phthisis justifiable in a general ward? The question is an interesting one, and it is exceedingly difficult to any agreement of opinion between hospital men and sanatorium enthusiasts. So long as the present inadequate provision for the isolation of consumptives is maintained, however, no general hospital can be justified in refusing to permit a phthisical patient to enter its wards. The crux of the tuberculosis problem, as Dr. Flick, of Philadelphia, points out in an able article contributed to the current issue of the *New York Medical Record*, is efficient isolation. The effectiveness of this measure in lowering the death-rate from consumption is indisputable. In Naples, where the principle of isolation was first honoured as far back as 1782, and was enforced for half a century, the death-rate dropped from 10 per thousand to 1 per thousand—a magnificent result when we take into consideration the fact that the Neapolitan hospitals of the time were not shining examples of what such institutions should be. The practice was to attempt isolation—isolation, that is, so far as the patient's fellow-men were concerned—by tending him apart from his family. As there were no sanatoria or consumption homes in those days, the phthisical patients were admitted into the general hospitals, certain portions of the ward being set aside for their use. Dr. Flick favours this practice on the ground that one of the chief advantages in opening up the general hospital to consumptives is the location of such hospitals in the centres of population near the homes of the people from among whom the stricken ones are to be taken. The poor are loth, he remarks, to permit their dying relatives to go beyond walking distance of their homes, and the sentiment which is at the bottom of their objection must be taken into account in any broad scheme of isolation. With this dictum most of us will agree. At the same time, the undesirability, for various reasons, of having consumptives in a general ward is very clear. Where the wards possess large balconies, and where such patients can be isolated in the open air, their admission may be justifiable on the argument put forward by Dr. Flick; but where such admission means their crowding into a general ward of patients whose chance of ultimate cure under the conditions that obtain in the institution are of the slightest, it is manifestly not in the interests of the hospital to encourage them. In how far the presence of such patients is a menace to the other occupants of the ward is a subject upon which it would be difficult to lay down any definite rules, since our views upon the infectivity of consumption in relation to attendants in consumption hospitals are so conflicting.

### THE DRUG MARKET.

While business in the drug market is slightly better than during the previous two weeks, the demand is still very quiet. Few changes in price of any importance have taken place. A meeting of British refiners of glycerin has decided to make no alteration in price at present; for the time being, therefore, the high prices which have been ruling for many months will be maintained. Cod-liver oil still exhibits a tendency towards lower prices, which is not surprising considering the smallness of the demand at present prevailing. There has been some improvement in the demand for citric acid, occasioned by the warm weather, and an advance in price is not improbable. The high price of Buchu leaves is well maintained. Jalap is still tending higher in value.

